# Early, Intense Rehabilitation Fails to Improve Outcome After Intra-Striatal Hemorrhage in Rats

**DOI:** 10.1177/15459683221137342

**Published:** 2022-11-16

**Authors:** Britt A. Fedor, Anna C.J. Kalisvaart, Shivani Ralhan, Tiffany F.C. Kung, Maxwell MacLaren, Frederick Colbourne

**Affiliations:** 1 Neuroscience and Mental Health Institute, Faculty of Medicine and Dentistry, University of Alberta, Edmonton, AB, Canada; 2 Department of Psychology, Faculty of Science, University of Alberta, Edmonton, AB, Canada

**Keywords:** stroke, rehabilitation, enrichment, intracerebral hemorrhage, neuroprotection

## Abstract

**Background:**

The formation and degradation of an intracerebral hemorrhage causes protracted cell death, and an extended window for intervention. Experimental studies find that rehabilitation mitigates late cell death, with accelerated hematoma clearance as a potential mechanism.

**Objective:**

We assessed whether early, intense, enriched rehabilitation (ER, environmental enrichment and massed skills training) enhances functional benefit, reduces brain injury, and augments hematoma clearance.

**Methods:**

In experiment 1, rats (n = 56) were randomized to intervention in the light (-L) or dark phase (-D) of their housing cycle, then to 10 days of ER or control (CON) treatment after collagenase-induced striatal intracerebral hemorrhage (ICH). ER rats were treated from 5 to 14 days after ICH. Behavior and residual hematoma volume was assessed on day 14. In experiment 2, rats (n = 72) were randomized to ER-D10, ER-D20, or CON-D. ER rats completed 10 or 20 days of training in the dark. Rats were euthanized on day 60 for histology. In both experiments, behavioral assessment was completed pre-ICH, pre-ER (day 4 post-ICH), and post-ER (experiment 1: days 13-14; experiment 2: days 16-17 and 30-31).

**Results:**

Reaching intensity was high but similar between ER-D10 and ER-L10. Unlike previous work, rehabilitation did not alter skilled reaching or hematoma resolution. Varying ER duration also did not affect reaching success or lesion volume.

**Conclusions:**

In contrast to others, and under these conditions, our findings show that striatal ICH was generally unresponsive to rehabilitation. This highlights the difficulty of replicating and extending published work, perhaps owing to small inter-study differences.

## Introduction

Intracerebral hemorrhage (ICH), caused by the rupture of vasculature in the brain, accounts for 10% to 20% of all strokes.^1^ This sudden extrusion of blood into surrounding tissue causes immediate mechanical trauma and delayed secondary injury. Although there are overlapping mechanisms of injury with ischemia, unique processes contribute to the protracted cell death observed in models of ICH.^2,3^ Pre-clinical studies illustrate progressive tissue loss occurring for weeks after collagenase-induced ICH,^2,4^ accompanied by decreases in cortical thickness and white matter atrophy. Degradation of the hematoma into cytotoxic components, such as heme and iron, causes inflammation, formation of reactive oxygen species, and a disrupted blood brain barrier.^5^ Similarly, intra-cerebral iron infusion alone causes progressive brain injury.^6^

To date, rehabilitation (rehab) remains our best treatment to promote recovery after stroke, yet much of our understanding of rehab comes from ischemic stroke, with more limited work exploring rehab exclusively after ICH.^7^ Animal^8^ and clinical^9^ evidence suggests that individuals recovering from ICH show greater early improvements than those recovering from ischemic stroke^10^; however, as acute treatment for ischemic stroke has improved, this finding has been challenged.^11^ There is a moderate body of animal research exploring rehab and recovery after ICH,^12-16^ and some of these studies have varied treatment parameters.^12,15,17^ From that limited work, it is difficult to optimize treatment protocols (e.g., timing and intensity of intervention) or to identify key underlying mechanisms. Thus, we must rely upon the known principles of learning and memory that can be harnessed to drive plasticity in the injured brain. Rehab utilizing optimal timing, specificity, repetition, intensity, and salience, all principles of experience-dependent plasticity, seem to best drive recovery after brain injury^18^ and it seems reasonable that the same should apply to ICH.

As with treatment parameters, the means by which rehab improves outcome have yet to be fully elucidated after ICH. Not surprisingly, rehab increases neurotrophic factors^19^ leading to the growth of spines and dendrites,^20^ synaptogenesis,^21^ and sometimes neurogenesis,^22^ (but see Auriat et al^20^). DeBow et al^12^ also discovered a neuroprotective effect with using constraint induced movement therapy (CIMT) following ICH. Despite the simplicity, running alone appears to be neuroprotective,^14^ but not all studies agree.^23^ Like CIMT, enriched rehabilitation (ER),^24^ a combination of skill training and environmental enrichment (EE), mitigated cell death after ICH while augmenting recovery.^25^ Both CIMT and ER align with several principles of experience-dependent plasticity, such as specificity, repetition, and intensity. As for timing, most ischemia and ICH studies begin rehab within the first 7 days after stroke,^13,20,25-27^ a time of heightened plasticity.^28^

Recently, accelerated hematoma clearance (mechanism unknown) with attenuated iron levels has been identified as a potential way by which rehab lessens injury (less oxidative stress) and neural dysfunction (less ionic dyshomeostasis).^29^ Drug-augmented clearance^30,31^ as well as surgical removal^32,33^ of the hematoma have been topics of interest for years,^34^ as reducing hematoma breakdown products should minimize secondary injury. For instance, pre-clinical studies administering lactoferrin show promise in augmenting hematoma clearance and improving behavior.^35-37^ It makes sense then that rehab’s known benefits (promoting synaptogenesis, etc.) might synergize with the neuroprotective and restorative effects of augmented hematoma clearance.

We must acknowledge that our treatment and mechanistic understanding of rehab, especially in the small ICH rehab sub-field, must be viewed in light of the fact that many pre-clinical stroke studies have shown interesting results, only to fail when re-tested or applied in a clinical setting.^38^ Numerous questionable practices, such as poor reporting, cherry picking data, and the use of small group sizes, all lead to overestimated effect sizes. Those issues, coupled with the more common failure to publish negative results, all contribute to the “replication crisis” in biomedical research.^39^ Thus additional high-quality studies are needed.

Here, we used a translationally rigorous design to explore whether treatment intensity and duration impact the efficacy of ER following ICH in rats. We were principally concerned about the neuroprotective effects of rehab, as we believe that this neuroprotective effect is biologically meaningful and at least partially underlies the better behavioral benefit reported in previous studies. As the basal ganglia is a common site of ICH in humans,^40^ we infused collagenase into the striatum to cause an ICH. This is a well-characterized^41^ and common model in ICH rehab studies, which have shown that rehab lessens secondary injury.^12,25^ Our first study assessed whether ER performed in the light or dark phase of the housing cycle altered rehab intensity and efficacy. Experimental work in ischemia suggests that rehab delivered in the dark leads to greater engagement in training and better outcomes than ER in the light phase.^27^ We used a hemoglobin assay to determine residual hematoma volume 14 days after ICH, as a comparable treatment was previously shown to accelerate hematoma clearance at 14 and 21 days after ICH in rat.^29^ Our second experiment explored whether increasing treatment duration (10 days vs 20 days of ER) provided superior behavioral benefit and neuroprotection when measured 60 days after ICH. We hypothesized that longer ER treatment would enhance behavioral and neuroprotective benefits owing to the ongoing secondary injury in this model.^2,3,6^

## Methods

### Animals

One hundred twenty-eight male Sprague-Dawley rats (250-300 g, ~2-4 months old) were obtained from Charles River (Saint Constant, Quebec). All procedures were approved by the Biosciences Animal Care and Use Committee at the University of Alberta (Protocol 960) and complied with Canadian Council of Animal Care Guidelines. Researchers adhered to the ARRIVE guidelines,^42^ except when blinding was not possible (e.g., during ER delivery and behavior assessment).

Rats were group housed 4 per cage in standard plexiglass cages (37 cm × 47 cm × 20 cm) with 2 standardized rat retreats (tubes) per cage in temperature- and humidity-controlled rooms, with lights on from 7 am to 7 pm (standard light cycle) or 7 pm to 7 am (dark cycle). In the week preceding behavior training, groups assigned to the dark cycle condition (all interventions completed in the dark), were transitioned to this schedule over a period of 4 days. Each day, the start of the light cycle was delayed by 3 hours (10 am-10 pm, 1 pm-1 am, 4 pm-4 am, 7 pm-7 am). To reduce animal stress, behavioral training was not performed until 2 days after transition. Rats assigned to ER groups were housed under the same conditions as control groups (CON), except during EE. Rodents were fed standard rat chow (Purina) with water ad libitum. Food and water were available ad libitum during acclimation phase, light cycle transition, pre- and post-surgery, and outside periods of behavioral testing. To reduce stress and familiarize animals to researchers, all animals received two 10-minute handling sessions with each researcher prior to the start of behavior training.

**Figure 1.**
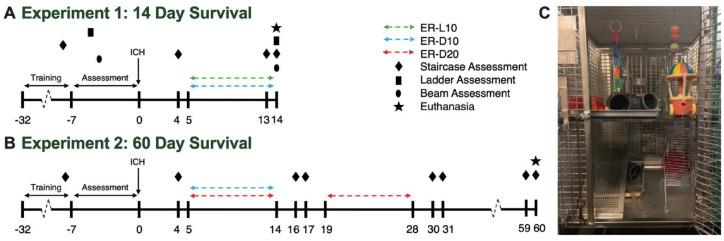
Experimental timeline. (A) Experiment 1: investigation of effects of ER on residual hematoma volume and behavior 14 days after collagenase-induced ICH. (B) Experiment 2: investigation of effect of altering ER duration (10 or 20 days) on lesion volume and behavior in the chronic phase of recovery after collagenase ICH. (C) Dual-level EE housing. Each level contained a running wheel for rodents to use at their leisure, as well as tubes for hiding, and novelty toys. To encourage exploratory behavior, food location was changed daily, and novelty items were changed twice weekly (eg, balls, chains, wooden blocks, cars). Cages were made of wire rungs to allow climbing between levels of the cage and included a ramp to allow more impaired animals to walk to upper level. Abbreviations: ER, enriched rehabilitation; ICH, intracerebral hemorrhage.

In experiment 1, rats (n = 56) were indiscriminately assigned to cages by animal care staff and were later randomized by cage (random number generator) to dark (D) or light (L) housing condition and transitioned to dark cycle housing appropriately (Figure 1A). Following behavior training and ICH induction, cages were further randomized to ER (ER-D10, ER-L10) or no treatment control (CON-D, CON-L) as noted in Table 1.

**Table 1.** Group Sizes, Exclusions, and Endpoints Analyzed.

Experiment	Group	Size	Exclusions	Endpoint analysis (n/group)
Experiment 1: 14 day survival	Dark Rehab (ER-D10)	16	Complete exclusions: 3 Partial exclusions: 3 (ladder)	Staircase: n = 13 Beam: n = 13 Ladder: n = 10 Hematoma volume: n = 13
	Light Rehab (ER-L10)	16	Complete exclusions: 3 Partial exclusions: 0	Staircase: n = 13 Beam: n = 13 Ladder: n = 13 Hematoma volume: n = 13
	Dark Control (CON-D)	12	Complete exclusions: 1 Partial exclusions: 1 (beam)	Staircase: n = 11 Beam: n = 10 Ladder: n = 11 Hematoma volume: n = 11
	Light Control (CON-L)	12	Complete exclusions: 3 Partial exclusions: 1 (beam)	Staircase: n = 9 Beam: n = 8 Ladder: n = 9 Hematoma volume: n = 9
	N = 56 Complete exclusions: n = 10 (7 failed to reach baseline behavior criteria; 3 technical error) Partial exclusions: n = 2 (beam—camera malfunction); 3 (ladder—camera malfunction)			
Experiment 2: 60 day survival	Rehab-10 (ER-D10)	24	Complete exclusions: 0 Partial exclusions: 1 (staircase), 1 (corpus callosum)	Staircase: n = 23 Lesion volume: n = 24 Corpus callosum: n = 23
	Rehab-20 (ER-D20)	24	Complete exclusions: 1 Partial exclusions: 2 (staircase), 1 (lesion volume), 2 (corpus callosum)	Staircase: n = 21 Lesion volume: n = 22 Corpus callosum: n = 22
	Control (CON-D)	24	Complete exclusions: 1 Partial exclusions: 0	Staircase: n = 23 Lesion volume: n = 23 Corpus callosum: n = 23
	N = 72 Complete exclusions: n = 2 (premature death) Partial exclusions: n = 3 (staircase—incomplete assessment), 1 (lesion volume—poor tissue quality), 3 (corpus callosum—poor tissue quality)			

Abbreviations: ER, enriched rehabilitation; D, dark; L, light; CON, control.

In experiment 2, all animals (n = 72) were transitioned to dark cycle housing (Figure 1B) as there was a small but non-significant trend that ER-D10 animals performed better at skilled reaching on day 14. Following behavior training and ICH, cages were randomized to 10 days of ER (ER-D10), 20 days of ER (ER-D20), or control (CON-D) condition (Table 1).

### Skilled Reach Training and Montoya Staircase Test

To encourage participation in behavioral training and testing, animals were food restricted to 90% of their free feeding weight with water available ad libitum in their home cages. Animals were trained on the staircase test^43^ for 4 weeks prior to stroke (5 days per week, 2 trials daily, 15-minutes each). Baseline assessment of skilled reaching and paw preference was determined on the last 3 days of training (average of the last 6 trials). Based on a priori exclusion criteria, animals that failed to successfully retrieve a minimum average of 9 pellets on at least one side were excluded from analysis but remained in the study and continued to receive treatment under their assigned protocol to prevent disruption to cage hierarchy and social dynamics.

All animals completed skilled reach testing prior to ICH (baseline testing), and after ICH on day 4, prior to the start of treatment. In experiment 1, skilled reaching was assessed over 4 trials on day 13 and 14 (2 trials/day, replacing the 7 am and 11 am rehab sessions on those days). In experiment 2, skilled reaching was assessed over 4 trials on days 16 and 17 (mid-point assessment) and days 30 and 31 (final assessment). Scores represent the average number of pellets retrieved across 4 trials.

### Beam Walking Task

Following completion of skilled reach training, animals were trained to cross a horizontal beam, as previously described.^3^ The final trial was video recorded and scored to determine baseline ability. Briefly, animals were scored as: 0 (rat fell off beam <10 seconds), 1 (could not place impaired limb on beam, stayed on >10 seconds), 2 (unable to cross beam but able to place impaired limb and maintain balance), 3 (able to cross beam but dragged impaired limb), 4 (able to cross beam, placed impaired limb on beam 1+ time), 5 (crossed beam with >50% slip rate on impaired limb), 6 (crossed beam with <50% slip rate on impaired limb), or 7 (crossed beam with 2 or less slips of impaired limb).^3^ Following all reach training sessions and reach assessment trials on day 14, rats completed one post-treatment beam walking assessment. Recordings were scored by a blinded researcher.

### Ladder Walking Task

Following completion of skilled reach training, rats were trained to cross a horizontal ladder apparatus with rungs spaced 1 to 5 cm apart, and scored as previously described.^44^ The final 4 trials were recorded to determine baseline walking ability. To avoid the ladder walking serving as rehab, post-ICH trials were only completed on day 14, after all skilled reaching and beam walking assessments were complete. Per a priori exclusion criteria, animals that failed to successfully cross the ladder a minimum of 2 times in either the baseline or day 14 assessments were excluded from analysis. The percentage of successful steps was calculated for the contralateral forelimb and averaged across all included trials:

$$\%\;successful\;steps=\frac{\#of\;successful\;steps}{\#of\;total\;steps}x100$$

### Surgery

For ICH, animals were anesthetized with isoflurane (graded induction to 4%, 2%-3% maintenance) and placed in a stereotaxic frame.^45^ A midline incision was made over the scalp to expose the surface of the skull and a burr hole was made 0.5 mm anterior and 3.5 mm lateral to Bregma,^46,47^ contralateral to the dominant paw (established during baseline reach testing). An infusion of 1.2 μL of 0.6 U bacterial collagenase (Type IV-S, Sigma) was injected 6.5 mm deep into the striatum over 5 minutes (26-gauge Hamilton syringe). The syringe remained in place for an additional 5 minutes to prevent backflow. The burr hole was then sealed with a small screw, and the scalp was closed. Temperature was monitored throughout the procedure via rectal probe and maintained at 37°C ± 0.5°C via heating pad. All animals received Marcaine (0.5 mL S.C., Pfizer Canada) for pain management at the incision site. Rats were provided with a wet mashed rat chow following surgery and recovery was monitored through daily weighing and health checks.

### ER and Training Intensity

Animals in ER training completed four 15-minute sessions of task specific training daily. Using a modified reaching apparatus,^13^ rats performed skilled reach training in individual plexiglass boxes. During these sessions, animals had access to ~200 reward pellets (Dustless Precision Pellets, Primate Purified Diet, Banana Flavor, 45 mg, Bio-Serv) in the well corresponding to their impaired limb. The start of each session was separated by 1.5 hours (7 am, 8:30 am, 10:00 am, 11:30 am). The weight of pellets consumed in each session was converted into the equivalent number of pellets retrieved during the session. Pellets retrieved was used to approximate rehab intensity.

Following completion of task specific training and daily feeding, rats assigned to ER were removed from their standard housing cages and placed in EE cages for 6 hours per day (1-7 pm). Animals completed EE within their light cycle, meaning that animals assigned to standard light cycle completed their EE during the light phase (experiment 1) and animals in the dark cycle completed EE in the dark (experiment 1, 2). Cages used for EE were dual level modified wire primate cages (71 cm × 71 cm × 89 cm) that included different types of wood shavings, small toys (changed twice per week for novelty), 2 running wheels, and a ramp between levels (Figure 1C).

### Hematoma Volume Assay

In Experiment 1, animals were deeply anaesthetized under isoflurane and euthanized by decapitation 14 days post-ICH. Blood volume of ipsilateral and contralateral hemispheres was measured using a spectrophotometric hemoglobin assay,^48^ as previously modified.^4,45^ To control for blood in the vasculature, hematoma volume (μL) was calculated as:

$$\begin{array}{c} hematoma\;volume=ipsilateral\;hemisphereblood\;volume-\\ contralateral\;hemisphereblood\;volume \end{array}$$

### Total Tissue Loss and White Matter Quantification

In Experiment 2, animals were euthanized at 60 days post-ICH and lesion volume was calculated.^4,49^ Briefly, animals were overdosed with sodium pentobarbital (Euthanyl, 100 mg/kg I.P., Bimedia-MTC) and transcardially perfused with saline followed by 10% neutral buffered formalin. Brains were extracted and fixed in formalin for at least a week, then transferred to a 30% sucrose solution for cryoprotection ~72 hours before cryosectioning. Coronal sections 20 μm-thick were taken every 200 μm, and stained with cresyl violet for lesion volume analysis and white matter atrophy.^49^ Fiji (ImageJ) software^50^ was used to quantify both total tissue loss (mm^3^) and white matter atrophy in the corpus callosum (mm^3^) by a blinded researcher. Total volume of tissue loss was calculated as:

$$\begin{array}{c} volume\;of\;tissue\;loss=volume\;of\;normal\;hemisphere-\\ volume\;of\;injured\;hemisphere,\\ volume\;of\;normal\;hemisphere=average\\ \left(area\;of\;hemisphere-area\;of\;ventricle-area\;of\;lesion\right)\\ x\;interval\;between\;sections\;x\#of\;sections\\ volume\;of\;injured\;hemisphere=average\\ \left(area\;of\;hemisphere-area\;of\;ventricle-area\;of\;lesion\right)\\ x\;interval\;between\;sections\;x\#of\;sections \end{array}$$

To assess white matter atrophy, the corpus callosum in each hemisphere was traced from AP: +1.5 to −0.5, with landmarks identified using the WHS rat brain atlas (v1.01, RRID: SCR_017124).^51^ This location encompassed 1 mm of tissue anterior and posterior to the site of collagenase injection and was reliably present in all brains. Volume of corpus callosum in each hemisphere was calculated as:

$$\begin{array}{c} volume\;of\;corpus\;callosum=average\\ (area\;of\;corpus\;callosum)\;x\;interval\;between\;sections\;x\\ \#of\;sections \end{array}$$

### Statistical Analysis

In experiment 1, group sizes were selected based on previous work.^29^ In experiment 2, group sizes were determined by a priori power calculation with reference to the most common marker of neuroprotection, and our primary endpoint, lesion volume. Using effect size and variance reported in similar work,^25^ group sizes of n = 24 were calculated to give at least 80% power to detect a 30% reduction in lesion volume with alpha set at .05.

All data was analyzed using GraphPad Prism (version 9.3.1 for Mac, GraphPad Software, San Diego, California). Baseline data was analyzed using ANOVA to assess group differences; when no baseline differences were found, all raw data was analyzed and reported. In experiment 1, two-way ANOVA was used to assess baseline reaching data, ladder walking success, rehab intensity, and hematoma volume. Three-way ANOVA was used to compare repeated measures reaching data. Beam walking data was analyzed using the Kruskal–Wallis test at baseline and day 14. In experiment 2, one-way ANOVA was used to assess baseline reaching data, hemispheric differences in corpus callosum, and lesion volume. Two-way ANOVA was used to compare repeated measures reaching data. When multiple groups were compared, ANOVA with Tukey’s or Sidak’s post hoc test was used. Data are reported as mean ± 95% confidence interval (CI), except for the beam walking data which are reported as median ± interquartile range (IQR).

## Results

### Mortality and Exclusions

See Table 1 for analyzed group sizes and exclusions; note that no animals were excluded based on their post-injury level of impairment.

There was no unexpected mortality in experiment 1. Ten animals were fully excluded from analysis, while an additional 5 animals were partially excluded and removed from analysis (beam test, n = 2; ladder walking task, n = 3).

In experiment 2, two animals died unexpectedly during surgery, presumably due to complications with anesthetic dosage. Seven animals were partially excluded and removed from analysis (skilled reaching, n = 3; lesion volume, n = 1; corpus callosum volume, n = 3).

### Experiment 1

#### Reaching Success

**Figure 2.**
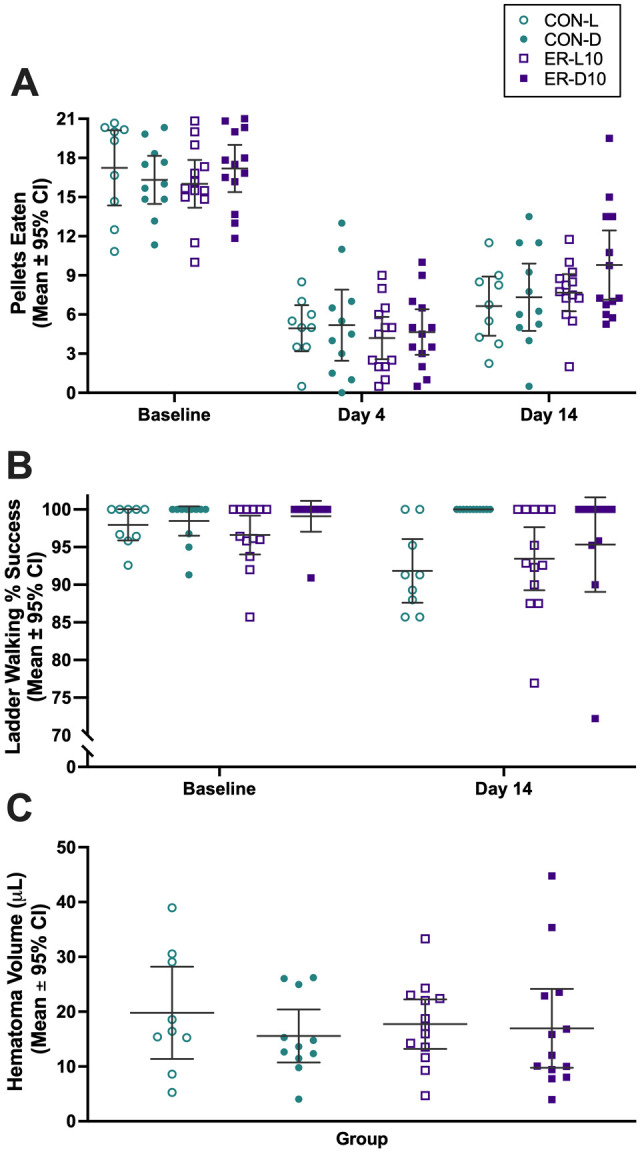
Results of experiment 1. (A) Reaching success in staircase task. All groups displayed notable impairment after ICH (vs baseline, persistent out to 14 days) but no effect of light cycle or treatment was detected. (B) Percent success of correct paw placement of contralateral forelimb on the ladder walking task. Three-way ANOVA detected a main effect of time and light cycle but not treatment. (C) Residual hematoma volume (μL) measured 14 days after ICH. Two-way ANOVA did not detect an effect of light cycle or treatment. Individual data points are shown along with the mean ± 95% CI. Abbreviations: ICH, intracerebral hemorrhage; CON, control; ER, enriched rehabilitation; D, dark; L, light.

There was no significant difference in baseline reaching abilities among groups (Figure 2A; light cycle, *P* = .8910; treatment, *P* = .8491; interaction, *P* = .2626). As anticipated, all groups were impaired on day 4 after ICH (time main effect, *P* < .0001) and impairment persisted to day 14 (*P* < .0001, two-way ANOVA with Tukey’s multiple comparisons test). Three-way ANOVA detected a main effect of time (*P* < .0001) but no main effect of light cycle (*P* = .3286), treatment (*P* = .6246), or any interaction (*P* ≥ .1371). While the ER-D10 group retrieved the greatest number of pellets on average at day 14, this result was not significantly different from the other groups (*P* ≥ .2171, two-way ANOVA with Tukey’s multiple comparisons test).

#### Rehabilitation Intensity

**Figure 3.**
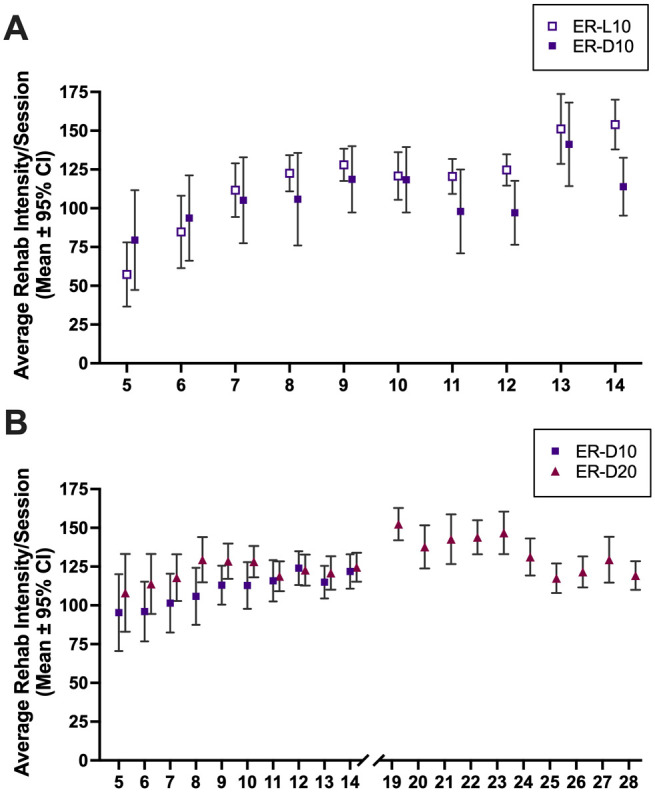
Rehabilitation intensity. Figures represent the average number of pellets successfully retrieved per training session on each day. (A) Experiment 1—rats in ER-L10 and ER-D10 groups completed 4 daily rehab training sessions days 4 to 12 and 2 sessions on days 13 and 14. (B) Experiment 2—rats in ER-D10 and ER-D20 groups completed 4 daily rehab training sessions days 4 to 14. Rats in ER-20 completed an additional 10 days of ER days 19 to 28. All data presented as mean ± 95% CI. Abbreviations: ER, enriched rehabilitation; D, dark; L, light.

Rats gradually increased pellets retrieved over time (time main effect, *P* < .0001) (Figure 3A). Light cycle did not alter reaching intensity (light cycle main effect, *P* = .2918), however an interaction was present (*P* = .0015). Sidak’s multiple comparisons test determined the groups were significantly different only at day 14 (*P* = .0167). Rats tended to reach somewhat more on the initial daily rehab session, and this declined over the 4 sessions within each day of rehab, presumably as they got satiated. This pattern of results varied modestly over days and groups, but without any meaningful pattern or obvious explanation for the failure to find behavioral improvements. The average number of pellets retrieved per trial from day 5 to 14 was 118.3 (95% CI: 97.71, 138.8) in the ER-L10 group and 107.1 (95% CI: 94.95, 119.3) in the ER-D10 group.

#### Beam Walking Task

All groups performed similarly at baseline (*P* = .5855) and at day 14 (*P* = .6729). Median beam walking score was 7 (7-7 IQR) in all groups at baseline. Nearly all animals received a perfect test score at day 14, suggesting that the beam walking assessment was not particularly sensitive to our injury. Median beam walking score was 7 (7-7 IQR) in ER-D10, ER-L10, and CON-D and 7 (6.5-7 IQR) in CON-L (data not shown). Rehab did not impact beam walking success.

#### Ladder Walking Task

Two-way ANOVA showed all groups performed similarly at baseline (light cycle, *P* = .1530; treatment, *P* = .7316; interaction, *P* = .3441). With no baseline differences, raw data were analyzed (Figure 2B). Three-way ANOVA detected a time main effect (*P* = .0095) and light cycle (*P* = .0060) but failed to detect a treatment effect (*P* = .4062) or interactions (*P* ≥ .0570). Generally, animals in the dark performed better than animals in the light. Owing to heterogeneity of variance in some of these data, we further analyzed it with several statistical tests (e.g., *t*-tests and non-parametric statistics). Post-hoc analysis revealed that the ladder task was not particularly sensitive to the stroke, and as such, we could not clearly assess whether rehab impacted walking success. For instance, there was no significant time effect (baseline vs day 14) for just the control groups, nor was there any evidence of benefit with either rehab treatment on the test day (statistics not shown).

#### Hematoma Volume

Residual hematoma volume at day 14 was ~17 μL on average (Figure 2C). Two-way ANOVA did not detect an effect of light cycle (*P* = .3874), treatment (*P* = .9073), or interaction (*P* = .5478). Thus, rehab did not impact hematoma resolution.

### Experiment 2

#### Reaching Success

**Figure 4.**
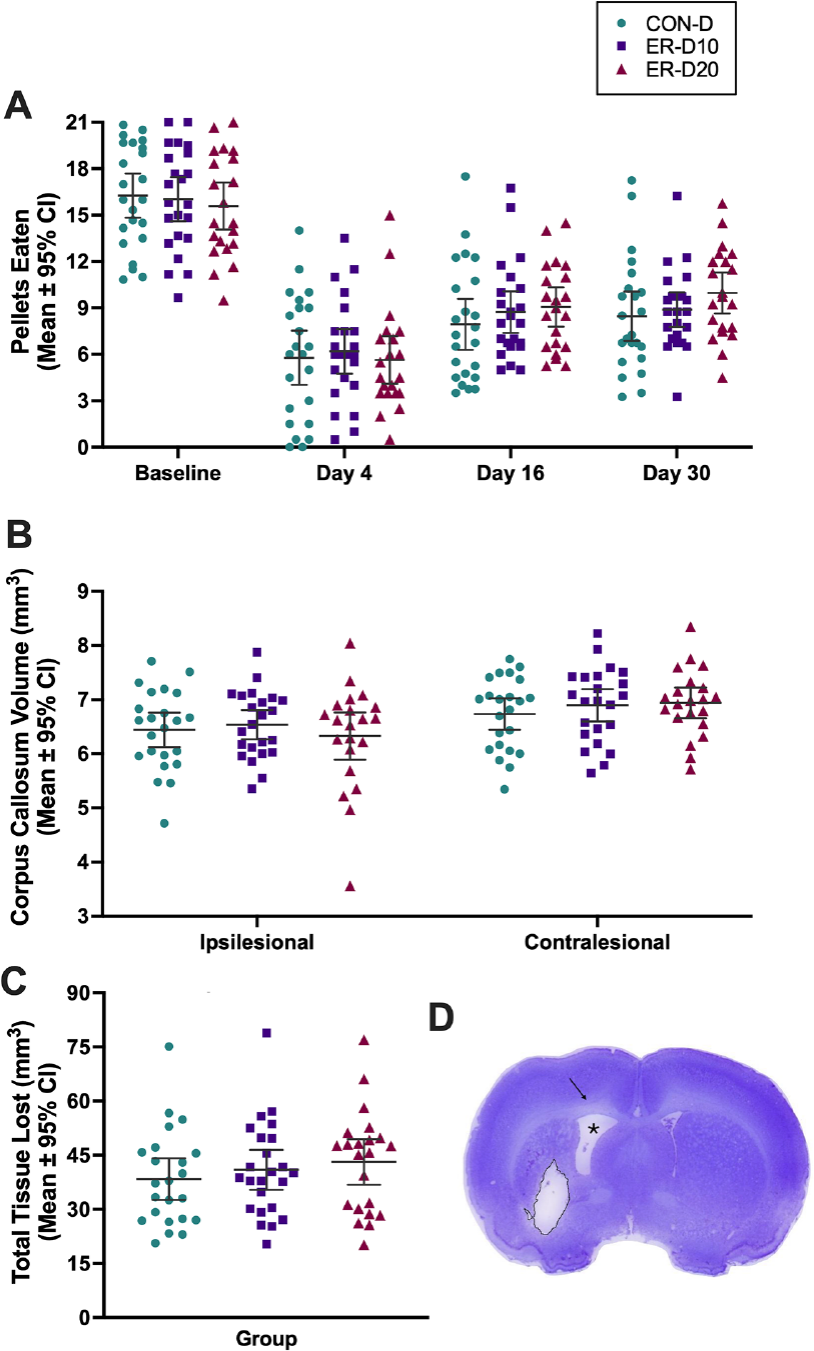
Results of experiment 2. (A) Reaching success in the staircase task was not improved by rehab. All groups displayed notable impairment after ICH (day 4). Multiple comparisons showed a main effect of time at all levels of comparison, except between day 16 and 30. We failed to detect an effect of treatment. (B) The volume of ipsilesional corpus callosum was smaller than contralateral; no effect of treatment was detected. (C) Lesion volume (mm^3^) measured 60 days after ICH; no effect of treatment was detected. (D) Coronal section of rat brain at 60 days displaying lesion cavity (outlined in black), ipsilesional ventriculomegaly (*), and atrophy of ipsilesional corpus callosum (black arrow). Subject in image had a total lesion volume of 38.8 mm^3^, approximately the average observed across groups. Individual data points are shown along with the mean ± 95% CI. Abbreviations: ICH, intracerebral hemorrhage; ER, enriched rehabilitation; CON, control; D, dark.

All groups performed similarly at baseline (*P* = .7906, Figure 4A). As anticipated, all groups displayed impairment after ICH (time main effect, *P* < .0001) but we failed to detect a treatment effect (*P* = .8275) or interaction (*P* = .3673). Tukey’s multiple comparisons test showed a main effect of time at all levels of comparison (*P* < .0001), except between day 16 and 30 (*P* = .1293). Thus, rehab did not improve reaching success, which mirrors the results from our 14-day survival experiment.

#### Rehabilitation Intensity

The average number of pellets retrieved per trial from day 5 to 14 was 110.2 (95% CI: 103.1, 117.4) in the ER-D10 group and 121.3 (95% CI: 116.3, 126.2) in the ER-D20 group and did not differ significantly on any day (*P* ≥ .3556; Figure 3B). The average number of pellets retrieved per trial from day 19 to 28 was 134.4 (95% CI: 125.7, 143.1). As in Experiment 1, rats tended to reach somewhat more on the initial daily rehab session, and this declined over the 4 sessions within each day of rehab, presumably as they got satiated. Again, this pattern of results varied modestly over days and groups, but without any meaningful pattern.

#### White Matter Quantification

The volume of corpus collosum remaining in the ICH hemisphere was significantly smaller than in the contralesional hemisphere (*P* = .001) in all groups. No effect of treatment was detected (treatment, *P* = .6898; interaction, *P* = .5495, Figure 4B).

#### Volume of Tissue Lost

All groups had significant tissue loss at 60 days as a result of cell death in striatum, white matter loss, and atrophy (Figure 4C). Treatment duration did not impact the volume of tissue lost (*P* = .5021), with an average of ~40 mm^3^ volume across groups.

#### Post-Hoc Pooled Analysis

**Figure 5.**
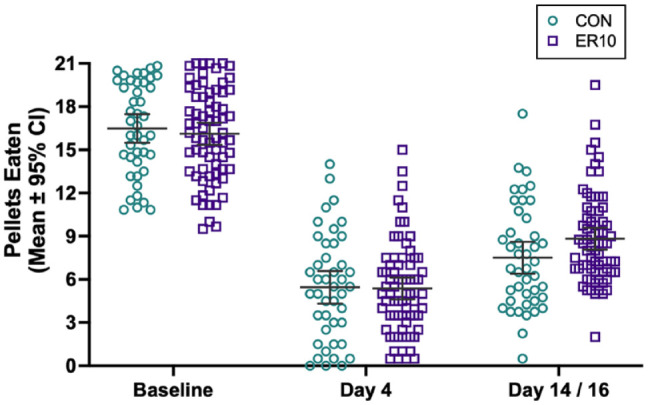
Post-hoc analysis of pooled data. Data from both experiments were pooled into ER10 (n = 70) and CON (n = 43) groups. A main effect of time (stroke-induced impairment) was readily evident on day 4 (10.85 fewer pellets retrieved [95% CI: 9.82, 11.88] vs baseline), and on day 14/16 (7.93 fewer pellets retrieved [95% CI: 6.96, 8.90] vs baseline). No significant effect of treatment was present (treatment effect size on day 14/16 was an improvement of 1.32 pellets retrieved [95% CI: −0.31, 2.95] vs day 4). Despite >99% statistical power to detect a 3-pellet difference, one level of the staircase task and presumably a minimum biologically meaningful effect, groups were not statistically different from each other after ICH. Individual data points are shown along with the mean ± 95% CI. Abbreviations: ER, enriched rehabilitation; CON, control; ICH, intracerebral hemorrhage.

Data from both experiments were pooled into ER10 (n = 70) and CON (n = 43) (Figure 5). A two-way ANOVA revealed a main effect of time (*P* < .0001), but no significant treatment effect (*P* = .5318) or interaction (*P* = .066). Compared to baseline, rats retrieved 10.85 fewer pellets (95% CI: 9.82, 11.88) on day 4, and 7.93 fewer pellets (95% CI: 6.96, 8.90) on day 14/16. Groups were not significantly different after ICH (day 4, *P* = .999; day 14/16, *P* = .146); treatment effect size on day 14/16 was an improvement of 1.32 pellets retrieved (95% CI: −0.31, 2.95) versus day 4. Thus, rehab did not notably change reaching success. This analysis had >99% power to detect a 3-pellet difference (one level of the staircase), a minimum effect we believe would signify biological importance.

## Discussion

Despite high-intensity training at a level and complexity comparable to previous work,^20,52^ three rehab protocols (ER-D10, ER-L10, ER-D20) failed to improve behavioral recovery after striatal ICH on our primary behavioral endpoint: skilled reaching. Contrary to previous work,^25,29^ rehab did not affect hematoma resolution or cell death. Our results highlight the difficulty of improving functional recovery of skilled movements after striatal bleeds, and the difficulty with reproducing or extending previous work. The latter undoubtedly contributes to translational failures.^39^

ER has been accepted as an effective intervention after experimental ischemic^24,26,27^ and hemorrhagic strokes.^13,20,25^ Likewise, other rehab methods, such as EE^53^ and running^14,54^ have been found beneficial. However, with these protocols, negative findings have been reported, and likely there are similar unpublished data. For instance, EE was of minimal benefit in several ICH studies^55,56^ and forced running was ineffective in one study.^23^ Studies also vary in the choice of behavioral tests and the timing of assessment; these factors may influence the apparent level of benefit observed (e.g., one test may be more sensitive to smaller treatment effects). Additionally, timing of assessment, differing sensitivity to lesion size, and degree of transference between rehab and skill assessment tasks may account for some of the reported differences in functional outcomes. Negative findings are generally dismissed owing to potential statistical (e.g., lack of power and bad luck), model (e.g., stroke severity), assessment (e.g., type and timing of testing), and treatment protocol issues (e.g., timing and intensity of rehab). Notwithstanding the aforementioned factors, the imprecise estimates of treatment effects, arising from small sample sizes, should not be underestimated for its contribution to study-to-study variability in outcome. In this study, we expected all of our ER treatments to improve skilled reaching given that comparable studies reported substantial effects in this ICH model^20,25,52^ and the autologous whole blood model.^13^ Since we used similar or identical methods (ICHs, rehab methods, and assessments) to past studies, one might surmise that statistical issues were at play, including bad luck or inadequate power. On the former, we made 4 independent tests of whether ER improves reaching and assessed both early (~14 days) and late (~31 days) into recovery. Further, we explored whether increasing ER treatment from 10 to 20 days would improve outcome. It did not. The net result yields no evidence for benefit despite multiple comparisons employing large groups sizes. Additional post-hoc analysis of ER and CON data pooled from both experiments (>99% power to detect a 3-pellet difference) showed only a slight non-significant trend in skilled reaching improvement in favor of ER when measured ~2 weeks after ICH, which is not reasonably expected to be of biological significance. As such, the publication of negative data is crucial^39^ to be considered along with other data in meta-analyses in order to accurately gauge treatment efficacy. As well, only with definitive evidence of efficacy can we truly make progress on confidently attributing cause to any mechanism(s) of action.

Our histological analysis showed typical ICH damage including: a lesion cavity, ventriculomegaly, and hemispheric atrophy. Contrary to earlier work^25,29^ and regardless of duration, ER failed to reduce injury. Use of ER also did not affect hematoma resolution in contrast to Williamson et al’s^29^ study, where ER beginning 1 week after ICH substantially accelerated hematoma clearance in 2 experiments. As previous work characterizing recovery after ICH demonstrated comparable behavioral impairment and tissue loss,^3^ we are confident that we induced a moderate ICH in these studies that should have been amenable to treatment (e.g., no floor or ceiling effects in the reaching task). Despite using ER during a well-established period of ongoing injury,^2,4,52^ the absence of behavioral and histological benefit in this study may ultimately stem from the lack of effect on hematoma clearance for whatever reason. If so, we surmise that when hematoma clearance is accelerated, injury is attenuated and behavior is improved.

Interestingly, we found no meaningful difference in the average rehab intensity between groups who completed intervention in the light or dark phase of their housing cycles. This differs from MacLellan et al,^27^ who found that following ischemia, rats completing ER during the dark phase were more engaged in rehab than those that completed the same task in the light phase. It should be noted that not all rehab protocols successfully replicate beneficial findings—in fact, the MacLellan study initially investigated rehab in the standard light cycle and failed to obtain benefit. As rats in the dark group completed ~230 successful reaches versus ~150 in the light, it was postulated that a certain threshold of intensity must be met to drive recovery. Rats in our experiments certainly exceeded this threshold, often reaching successfully more than 400 times/day. Perhaps treatment-induced recovery after ICH differs from ischemia, including that high therapeutic intensity may negatively impact rehab efficacy. Location is also likely a factor, with striatal injury perhaps less amenable to rehab than injury involving the cortex.^27^

Many consider earlier interventions to be more beneficial to recovery than delayed interventions,^26^ with early mobilization being a common recommendation in clinical guidelines.^57^ However, the best timing, intensity, and frequency of intervention is yet to be elucidated. Evidence from the AVERT clinical trial found that higher dose, early, intense out of bed mobilization in the first days after stroke may reduce odds of favorable outcome.^58^ Subgroup analysis showed this effect to be most prominent for those with severe stroke and ICH,^58^ with further analysis showing that increased frequency (number of mobilization events) but not increased intensity (time out of bed) was associated with improved outcomes.^59^ While other pre-clinical studies have reported benefit of various rehab paradigms beginning as early as 1 day after ICH, many fail to report the key parameters necessary to interpret treatment dose, such as treatment frequency and intensity. This poses a challenge to researchers and clinicians attempting to translate pre-clinical findings into clinical success. Here, we utilized a well-established therapy, with minor modifications to previously published work. Animals actively engaged with this treatment (both in reaching and EE) and overall achieved a high dose of therapy beginning at a time thought to be safe, yet we were unable to find comparable results to similar studies. Perhaps differences among treatment protocols (e.g., a day 5 vs day 7 start; 6 hours EE vs 10 hours EE; training sessions spaced 1.5 hours vs 2 hours apart; treatment in light vs dark) underlie study outcome differences (e.g., longer EE may provide additional benefits). However, these findings likely speak to the “replication crisis” of biomedical research, where reducing variability in our groups to achieve higher internal validity, often comes at the sacrifice of external validity and therapeutic translation.^60^ If so, these results speak to the finicky and challenging nature of translating therapeutic parameters to work in humans.

## Conclusion

Despite group sizes up to 3 times the average typically reported in pre-clinical ICH neuroprotection research (n = 8),^38^ our results show that intense ER beginning 5 days after ICH failed to improve outcome when assessed at day 14, during a period of ongoing cell death, and out to day 60, after cell death is believed to be complete. These results underscore the importance of studying rehab after ICH, and the necessity for future work to be conducted with higher power and factors that may impact translation in mind.
